# New model of sports tourism with sustainable tourism development to increase tourist arrivals in Central Aceh Regency, Indonesia

**DOI:** 10.3389/fspor.2024.1421363

**Published:** 2024-07-11

**Authors:** Yoki Afriandy Rangkuti, Heny Setyawati, Mugiyo Hartono, Taufiq Hidayah

**Affiliations:** Postgraduate Faculty of Sports Science, Universitas Negeri Semarang, Semarang City, Indonesia

**Keywords:** new model, sports tourism, increase tourist visits, Central Aceh, Indonesia

## Abstract

**Introduction:**

This study explores the development and implementation of a new sports tourism product called “Run H2O Ride” as a strategy for sustainable tourism in Indonesia.

**Methods:**

The research employs a research and development (R&D) methodology, focusing on identifying potential issues, conducting literature reviews, designing the product, validating the design, and undergoing product development. A combination of discussion group forums (FGD) and expert judgment decisions was used to design the new sport tourism model.

**Results:**

The effectiveness of the model was assessed through limited product tests, main product tests, and operational product tests involving respondents from the local community and tourists. The results indicate that the “Run H2O Ride” model has been well-received, with positive feedback on its suitability and effectiveness in attracting tourists and enhancing the local economy.

**Discussion:**

The study concludes by recommending further research to refine the model and emphasizes the importance of local government support and community participation in sustainable tourism development. Theoretical implications highlight the significance of sports tourism events in promoting tourism, while practical implications suggest alternative solutions for increasing tourist visits and improving destination image. Policy recommendations for local governments are proposed to adopt and implement sports tourism events, aligning with long-term development plans for regional tourism growth.

## 1 Introduction

The creation of jobs, the distribution of wealth, and the development of local culture are recognized economic benefits of tourism at the global level. Being the world's largest financial sector (1), tourism stimulates exports, creates jobs, and improves the lives of millions of people (2). Radicchi (3) states that tourism is one of the economic sectors often discussed from several points of view used to measure the nature of the sector. Contemporary tourism is evolving and becoming the foremost industrial sector in the world due to its positive social, cultural, and economic effects. Many countries benefit from this industry in meeting the demands of tourists (4, 5). Brykova et al. and Alimov et al. (2, 6) state that because it plays a direct role in the creation and growth of a nation's tourist activities, the degree to which a country realizes its tourism potential determines how successful the tourism industry in that nation will be. In general, the tourism business is part of the economic revival of many developing countries (7, 8).

Indonesia is one of the developing countries, so Indonesian tourism is now predicted to be able to replace state income from the mining sector, which has been the main sector to date, because tourism is a labor-intensive sector that has a direct influence on the community (9–13). Economic development is a complex procedure that includes significant adjustments to social and economic structures, including eradicating poverty, reducing inequality, and resolving unemployment in the context of economic expansion (13–15). To create resilience in communities, regions, and countries, sports events have proved to benefit communities and hosts in many ways, including creating jobs and a better perception of the location (16).

Sporting events are an important part of tourism products that are utilized to optimize economic impact and improve the image of the host destinations (17) and effectively reduce poverty in some local communities (18) through the creation of new jobs (19). These events function akin to a symbiotic mutualism regarding tourism and sports revenue (20). They have even become an industry in building economic and social benefits for countries around the world (21, 22). Ferranti et al. (23) highlight the positive appeal felt, especially in developing countries. The positive impact of sports tourism events must also be felt by rural local communities to build equality in developing countries (24). In addition, sustainable tourism development can be integrated with providing employment and good infrastructure development (25).

This clarifies what Carr et al. (26) expressed about the principles of long-term sustainability and natural resource management being able to support all forms of tourism, including the planning, execution, and oversight of local community tourism. Researchers have previously published journal articles and/or seminar manuscripts on a variety of topics related to local community tourism (26–31). This early work supports the development of the economic welfare of local communities and the development of tourism to enhance social, cultural, and place identities for surrounding communities (32–35). As revealed by Han et al., Kim et al., and Lee (36–38), sustainable tourism development is important in meeting the needs of tourists and adding to the economy of the surrounding community so that the quality of life improves for all.

The debate on the significance of sporting events, therefore, centers on the strategy of promoting tourism, by either exploring sports tourism destinations, or focusing on sports tourism heritage events (39). Researchers in the field of marketing assert that the image of the destination is very important (40). Moreover, Jenkins (41) suggests that leveraging sporting events (legacy) is preferable to merely hosting them. Accurate curation of the future tourism segmentation is crucial (42). Specifically, both central and local governments are encouraging tourism activities in Central Aceh District, Aceh Province, Indonesia, by promoting them through sports tourism events. Unfortunately, there have not been many previous studies that deeply examine the development of sports tourism by combining these two elements: exploring tourist destinations utilizing natural beauty and sports heritage. Therefore, this study aims to accomplish the following: (1) to design a new sports tourism model suitable for organizing the “Tour Delut Tawar” event in Central Aceh District, Aceh Province, and (2) to test the suitability and effectiveness of the new product model to increase future tourist arrivals. This research is significant as a preliminary design related to the development of new sports tourism products in Aceh Province. It also aims to support the Aceh Provincial Government's Long-Term Development Plan 2023–2027, which calls for new sports tourism products to be developed in the sports industry, contributing to regional foreign exchange income and improving the community's economy (43).

## 2 Conceptual framework

### 2.1 Sports tourism

Tourism and sports are complementary components and intersect with culture, impacting social behavior (44). Many developed and developing countries have established and popularized sports tourism (11). Sports are a common motivation for tourists in going on tourist trips (45). González-García et al. and Preuss (46, 47) affirm that sports tourism could be interpreted as activities carried out while traveling or staying in places outside one’s usual environment. Hinch and Higham (48) define the idea of sports tourism as a short trip centered on sports away from home, where tournaments involve the uniqueness of each region, physical prowess, and games played according to certain rules. Chang et al. and Gibson (49, 50) state that sports tourism is recreation-based travel that takes individuals outside their home community to participate in and watch sports activities. Dauter (51), Fries (52), Gilman and Huebner (53), Hallmann et al. (54), and Nelson et al. (55) have stressed that a lot of people's lives these days revolve around leisure and physical activity in general, and that leading an active lifestyle is beneficial to one's health and wellbeing, both passively by going to sporting events as a spectator and actively by playing sports on vacation or even traveling specifically to attend sporting events. Three categories of sports tourist activities are widely acknowledged: history or nostalgic sports tourism, sports event tourism, and active sports tourism (56). Malchrowicz-Mosko and Munsters (57) and Woodham (58) emphasize tourist intentions are divided into two basic forms: active sports tourism, which is based on elite athletes through physical participation and competition, and passive sports tourism, which is based solely on attending sporting events as spectators and visiting new places.

The tourism industry is now starting to use sports and local culture as a means of promotion to impact economic expansion by attracting visitors (59). Misener and Mason (60) state that public officials believe that sports and sporting events can catalyze local development. Tourism researchers suggest that sports tourism events coincide with elevating the traditions of local communities as a resource for developing future tourism initiatives (61). Maintaining the sports tourism sector and gaining sympathy from local communities is considered significant (49). In addition, the poor image of a destination (62) is a significant factor influencing residents’ support for developing sports tourism in their area (63). The surrounding and local communities will feel the benefits and provide support in realizing sustainable tourism in their area (64, 65). Conversely, marginalizing local people from tourism expansion will deplete the chances of success and impact socioeconomic inequality (49, 66).

### 2.2 Indonesia's sports tourism potential and master plan

The development of the tourism industry is one of the strategic steps that can be taken to support the National Sports Grand Design (DBON) program. This design is a master plan that outlines the national sports coaching and development policies’ specific directions to be implemented effectively, efficiently, superiorly, measurably, systematically, accountably, and sustainably, especially in the fields of education, recreation, achievement, and the sports industry based on science and technology (67). One of the goals is to advance the sports-based national economy (49). Zulfikar et al. (68) state that sports as a tourist attraction have made tourism and the sports industry cater to tourists seeking a sports experience. Furthermore, the DBON states that the sports industry is directed to developing sports tourism (69). The tourism industry has begun to recognize the existence of sports tourism as a part of tourism activity where tourists are directly involved in sports activities or see sports-related activities (70). Meanwhile, Bangun (71) and Lagarense et al. (72), claim that sports tourism is the new paradigm for the growth of travel and tourism in Indonesia.

Indonesia is an archipelagic country consisting of thousands of islands (73) and has a variety of tribes, languages, customs, and cultures. UNESCO has begun documenting the culture of all countries in the world as World Cultural Heritage (World Heritage) (74–76). Ramón-Cardona et al. (74) state that understanding the significance of specific cultural and natural sites to enhance their conservation and raise awareness can encourage tourism and benefit the surrounding area. Gański (77) states that tourists are attracted mainly by the rich historical and cultural heritage. Dladla et al. (78) state that cultural heritage tourism can attract many tourists because of its significance to society. Culture is very close to sports (79, 80). Irfan et al. and Yu et al. (81, 82) state that public demand for sports cultural tourism will decline along with the combination of the development of conventional sports cultural resources and sports cultural tourism, which will ultimately push the boundaries of traditional fashion and impact the development of a quality sports tourism sector. Hinch and Higham (48) state that sports tourism shows that the evolution of sports can directly impact the progress of tourism. Perić (83) states that the impact of culture on sports tourism is related to moral and social changes as well as the economy of a country. In some situations, a country's cultural and environmental effects are related to the positive impact on state services to its people, creating tourism prospects in that country (84). In addition, tourism researchers suggested that these sports tourism events use sports legacy as a resource to develop new tourism strategies aimed at drawing sports visitors (61).

The Gayo tribe is one of the ethnic groups in Aceh Province and spreads mostly in Central Aceh Regency, located on the westernmost island of Sumatra. This tribe contributes to the diversity of tribes in Indonesia. The Gayo tribe is an indigenous group of people from the Gayo highlands, which is the area around Lake Laut Tawar at an altitude of 1,500 m above sea level, so this area is known as the land above the clouds. The sports heritage event for the Gayo tribe is known as “Pacu Kude”, commonly known as the sport of horse racing, and it has long been contested during the Dutch colonial era (85). This is stated in the Indonesian Ministry of Education and Culture Decree regarding intangible cultural heritage. In general, the older the show, the more embedded it becomes as part of the region's heritage and the stronger the brand in commercial terms (86). Cultural heritage is important in local tourism development strategies because it attracts visitors, preserves cultural identity, and encourages regional economic growth (78). Sustainable tourism management is necessary for this tourist destination, which is one of the world's biggest industries with extensive cultural heritage that offers several resources to raise the community's standard of living (87).

However, the current flow of globalization threatens the existence of sports heritage, which should be able to integrate perfectly into the sports tourism industry. Even sports themselves are also eroded by globalization. As expressed by Higham et al. (88, 89), a sport reflects individual identities that have been confounded and national identities that the forces of globalization have eroded. Higham (89) states that the development and hybridization of sports and the growth of virtual sports and online gaming represent an ongoing evolutionary route. Even more dramatic is the idea that greater access to space will give rise to a new generation of sports, some already under development, such as solar-powered moon sailing and groundbreaking, zero-gravity sporting facilities (90). It is not impossible that the threat of sports tourism in the future will also lead to this. Kuzior et al. (91) state that globalization and the Industrial Revolution triggered increased competitiveness in the market, which necessitated a paradigm shift in tourism business models.

There is greater competition in the sports tourism business today after the pandemic, where each region competes to have a good image. The dark history of war conflicts from 1976 to 2005 still affects the bad image of this area for tourists who want to visit. The growth of this industry in Indonesia is intrinsically a political activity based on various factors and interests (92). The transformation of sports tourism must be based on the innovation matrix of local governments, which will allow tourist destinations to emerge from the crisis and solve environmental, economic, social sustainability problems (93–95), and political impact (68). As revealed by Su et al. (96, 97), tourists frequently view a location with an exceptional reputation as more reliable, valid, and competitive. Some destinations are tourism hubs. Therefore, authorities can distinguish tourist destinations from similar destinations by positioning them as unique and competitive, making them more attractive (98). Chalip et al. and Chalip and McGuirty (99–101) state that marketers have focused on organizing sports events as a strategy to increase the image of each region's destinations and differentiate their tourism products. Although Hassan (102) states that competitiveness is the capacity to develop and incorporate value-added products while preserving resources and a position in the market relative to rivals, it also refers to a destination's relative capacity to satisfy visitor needs on elements of the tourism experience that tourists consider important (103, 104), which includes synergetic elements addressing visitors’ needs, wants, and desires, given their time and budget constraints (105, 106). Bruhn and Rohlmann (107) state that sports are now more important and interesting to all social classes. Preuß (108) defines sports brands as a distinct image deeply ingrained in the minds of fans and other reference groups, according to a social psychology phenomenon applied to sports brands.

In addition, natural wealth in Central Aceh Regency has not been fully utilized as an object of sports tourism to attract visitors. As revealed by Wijaya et al. (109), Pakaya et al. (110), Rahmafitria and Misran (111), Mallen and Adams (112), and Rusyanto Fitriantono and Kristiyanto (113), there are seven requirements indicating natural potential in Indonesia that can be met to create natural destinations; the following factors affect geography: (1) topography and landforms; (2) climate and weather; (3) rock material; (4) Geographical Location; (5) water; (6) flora; and (7) wildlife. If promoted effectively, sports tourism and nature can exploit a wide, sports-friendly audience willing to participate in exciting tourist experiences (114). Thus, tourism highly depends on a destination's environmental/natural and cultural attractiveness (97, 115, 116). According to Hallmann et al. (54), the sports tourism industry makes it possible to take advantage of this opportunity by configuring the right offerings for the purpose and giving visitors the illusion of adventure and experience. However, this has not been done by the Regional Government through the Culture and Tourism Office and the Youth and Sports Office in Central Aceh Regency because it only follows the trend of the image of other regions that have long built and carried out their sports tourism activities. One of these trends is organizing events internationally and spending a lot of money but getting minimal results (117, 118). Thus, the target number of visitors and participants who attended the “Tour Delut Tawar” event was not achieved, as presented in Table 1.

**Table 1:** Data on the results of the 2022 “Tour Delut Tawar” event.

No.	Targets and results of implementation	Target	Result
1.	Tourist	2,000 people	10 people
2.	Participants	100 racers	11 racers
3.	International participants	10 countries	3 countries

Source: own elaboration.

Indications of failing to achieve targets and results in the event are due to only including bicycle races as an attraction. Several authors have drawn attention to the inefficiencies in the industry caused by the lack of collaboration between sports and tourism organizations (42, 119–123). Tchetchik et al. (105) emphasize that this tourist attraction is the main tourism product that provides various goods and services to attract tourists. This is an important reason for the existence of tourist destinations and a major pull factor for sports tourism development purposes (124–126). Febrianto et al. (127) state that sports tourism events should improve the community's economy because this competition can invite many people to attend and watch the race. Furthermore, it improves the economic welfare of residents (128).

This research is an original research work that fills the gap in the sports tourism literature with product development based on several stages of testing, consisting of limited trials, main trials, and operational tests conducted on tourist visitors in Central Aceh Regency. A preliminary study, especially in Aceh Province, investigates a new model of sports tourism in organizing “Tour Delut Tawar” events, highlighting the gap between the expectations and reality concerning results and targets when organizing events. This research offers a unique and valuable contribution that has never been explored before, hence contributing significantly to research on sustainable tourism in the future.

## 3 Methods

This study employed the research and development (R&D) methodology, a process used to produce certain products and analyze their effectiveness (129, 130). The R&D followed the Richey and Klein procedure improvement methodology at level 4 investigation (131, 132). The steps involved in this research process are identifying potential issues, literature review and data collection, product design, design validation, and product development. The product has also undergone restricted testing, a main trial, an operational trial, a second product revision, a third product revision, and a first trial. Dissemination and implementation are the last steps.

**Figure 1:**
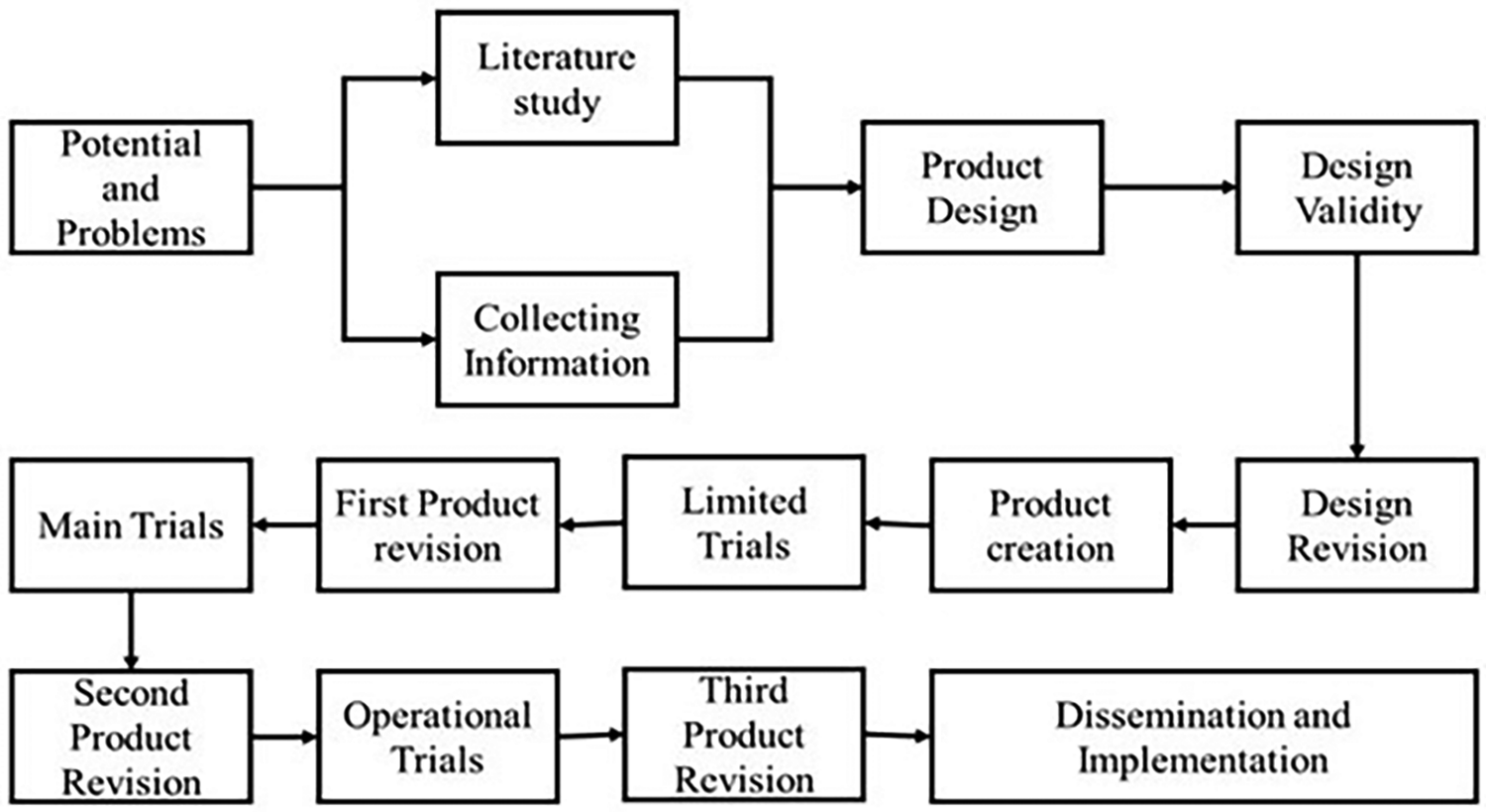
Procedure of product development chart.

Two methods were used to design a new sports tourism model, namely, a discussion group forum (DGF) and expert judgment decisions. The DGF consisted of a closed questionnaire involving experts from academia, the tourism culture office, and the youth sports office as representatives of the local government, totaling 20 people. After that, a preliminary study of the initial product design was conducted, focusing on the types of sports included in the model, route selection, and mileage, carried out in the field. The data were of the questionnaire instrument analysis rubric kind. Data were gathered in multiple phases to achieve predefined goals, including product design, design stage, design validation, product design revision, and product production. During the design stage, the research team kept in close contact with experts to choose a product design that best fits the needs analysis findings. The collection of research implementation procedures can be seen in the following (Figure 1) flowchart.

Expert judgment decisions, collection methods, and content validity all contribute to the validation of the research data. In addition, external validation is conducted using the Likert scale (4-point) and product moment correlation with 20 samples and 10 statement items. The Cronbach alpha reliability test was used to analyze reliability. The answers were synthesized and statistically processed using SPSS Statistics, version 26 (IBM Corp., Armonk, NY, USA) (133).

The overall sample size in the study consisted of 105 respondents. This study used a mixed methods sampling approach, or combination sampling method (134, 135), which consists of purposive sampling (direct designation) and random sampling (random selection) based on specific criteria related to individuals who have prior experience in running, swimming, and riding. The use of a combination of purposive sampling and random sampling is very relevant in research (136) that requires the inclusion of individuals with specific skills or characteristics while also aiming for generalizable results. All respondents were asked to fill out a questionnaire via Google Forms and submit their answers during the interval from 25 February to 24 March 2024.

The questioners were distributed and implemented in limited trials, main trials, and operational trials as the subsequent phases to determine the viability of research products (137). In determining the sample size, calculating the standard error (SE) involves dividing the standard deviation (SD) of the dependent variable by the square root of the previously selected sample size (n). If the result is below 2.0, it indicates that the number of samples is sufficient, as it proves to be homogeneous and representative of the population. For practicality, the target was to recruit 15 respondents for limited trials, 30 respondents for main trials, and 60 respondents for operational trials. The exploratory method was used by understanding that an analysis involving planning, creation, and assessment was the primary aim of planning and development research. All respondents in this study had agreed to participate and signed the consent form.

## 4 Results

Based on the initial information collected through the DGF, as presented in Table 2, it was agreed that in the selection of a new model of sports tourism to increase tourist visits, the sport of triathlon would be modified with the sports heritage of the Gayo tribe. This new sport, named “Run H2O Ride”, combines running, swimming, and horse riding. In addition, rules, regulations, routes, and mileage were adjusted based on the needs analysis of travelers, particularly to accommodate family-oriented segments. Previously known sports tourism events centered around activities like marathons and triathlons (137–139). These triathlons generally involve swimming, cycling, and running (112, 140) with stringent race rules and regulations (141–146) that can exclude many tourists from participating.

**Table 2:** Discussion group forum.

No.	Aspects	Answer: Yes (person)	Answer: No (person)
1.	Development of sports tourism with sports heritage	19	1
2.	Tourism that can attract the wider community	18	2
3.	Sports that can attract the wider community	20	0
4.	Developed “Ride H2O Run” for sports tourism	20	0
5.	Sports tourism sites in Pante Menye beach	20	0

Source: own elaboration.

**Figure 2:**
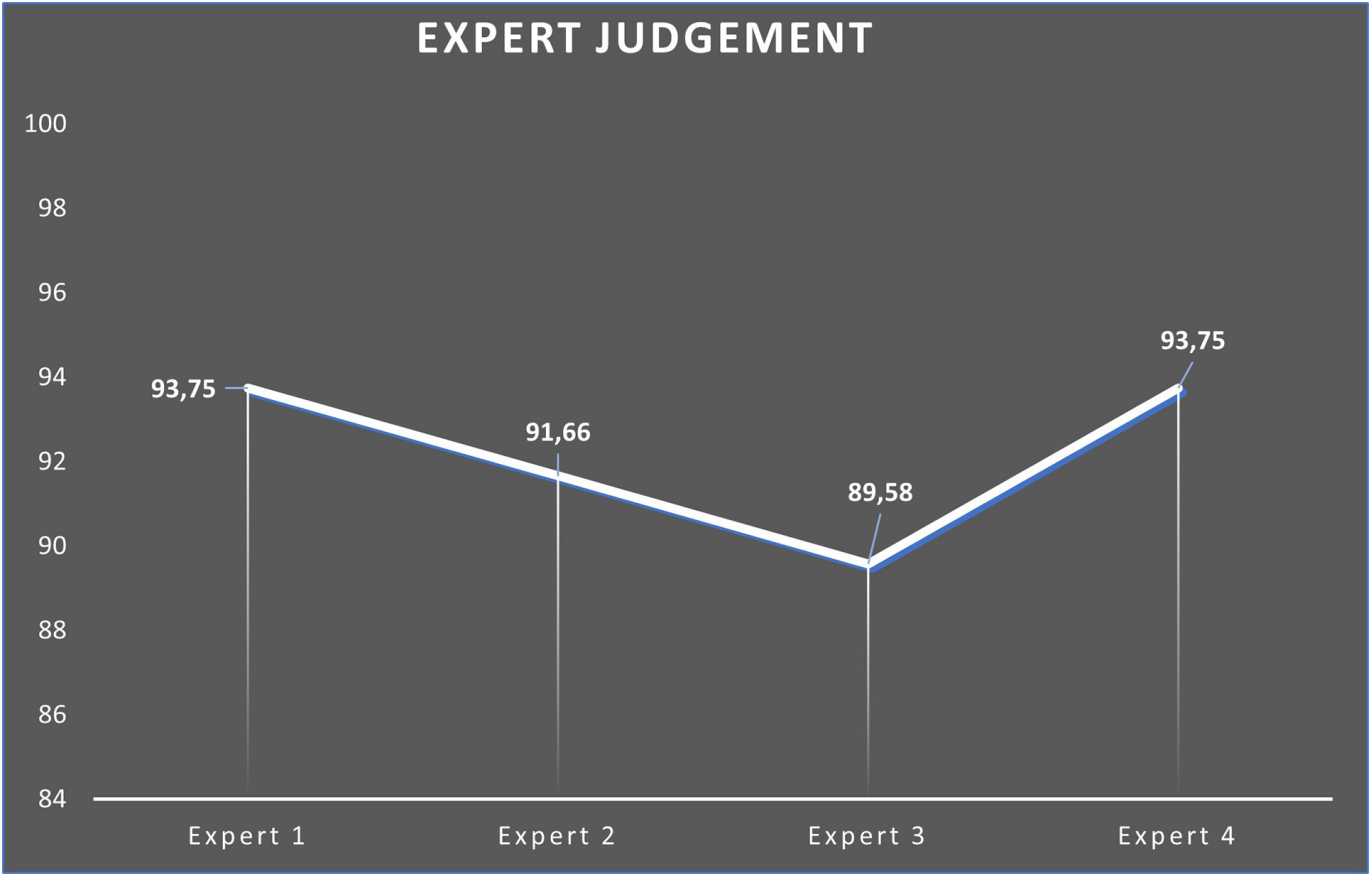
Expert assessment.

A literature review was done to develop an effective product design. Industry requirements and the results of the literature review were used to create products. All development concepts went through the research stages specified in the research methodology. Subsequently, the product was validated by experts using a Likert scale with four ratings (147) as presented in Table 3. Four experts participated in the expert validation product assessment, which was conducted using descriptive statistical analysis. The results of the assessment are as follows (Figure 2).

**Table 3:** Qualification assessment of the “Run H2O Ride” rubric by experts.

No	Statement	Expert 1	Expert 2	Expert 3	Expert 4
1.	The need for the development of new sports tourism models at the “Tour Delut Tawar” event	4	4	4	4
2.	The type of sport chosen for the development of the sports tourism model is the “Run H2O Ride”	4	4	4	4
3.	The suitability of the type of sport chosen for the development of a sports tourism model	4	4	3	4
4.	The mileage used for running 2 km	4	3	4	3
5.	Mileage used for swimming 500 m	4	4	3	4
6.	The mileage used for horse riding is 3 km	4	4	4	3
7.	Site selection of the development of “Run H2O Ride”	4	4	3	4
8.	Routes used for “Run H2O Ride”	3	3	3	4
9.	Equipment of facilities and infrastructure used	3	3	3	3
10.	Race rules used for the “Run H2O Ride” sports tourism model	3	3	3	4
11.	The need for the development of a sports tourism model	4	4	4	4
12.	Suitability of the model to the characteristics of the surrounding community and tourists who come to visit	4	4	4	4
Total amount		45	44	43	45

Value 4 = VP, very precisely, Value 3 = E, exactly, Value 2 = I, inaccurate, Value 1 = VI, very inaccurate.

Source: own elaboration.

**Figure 3:**
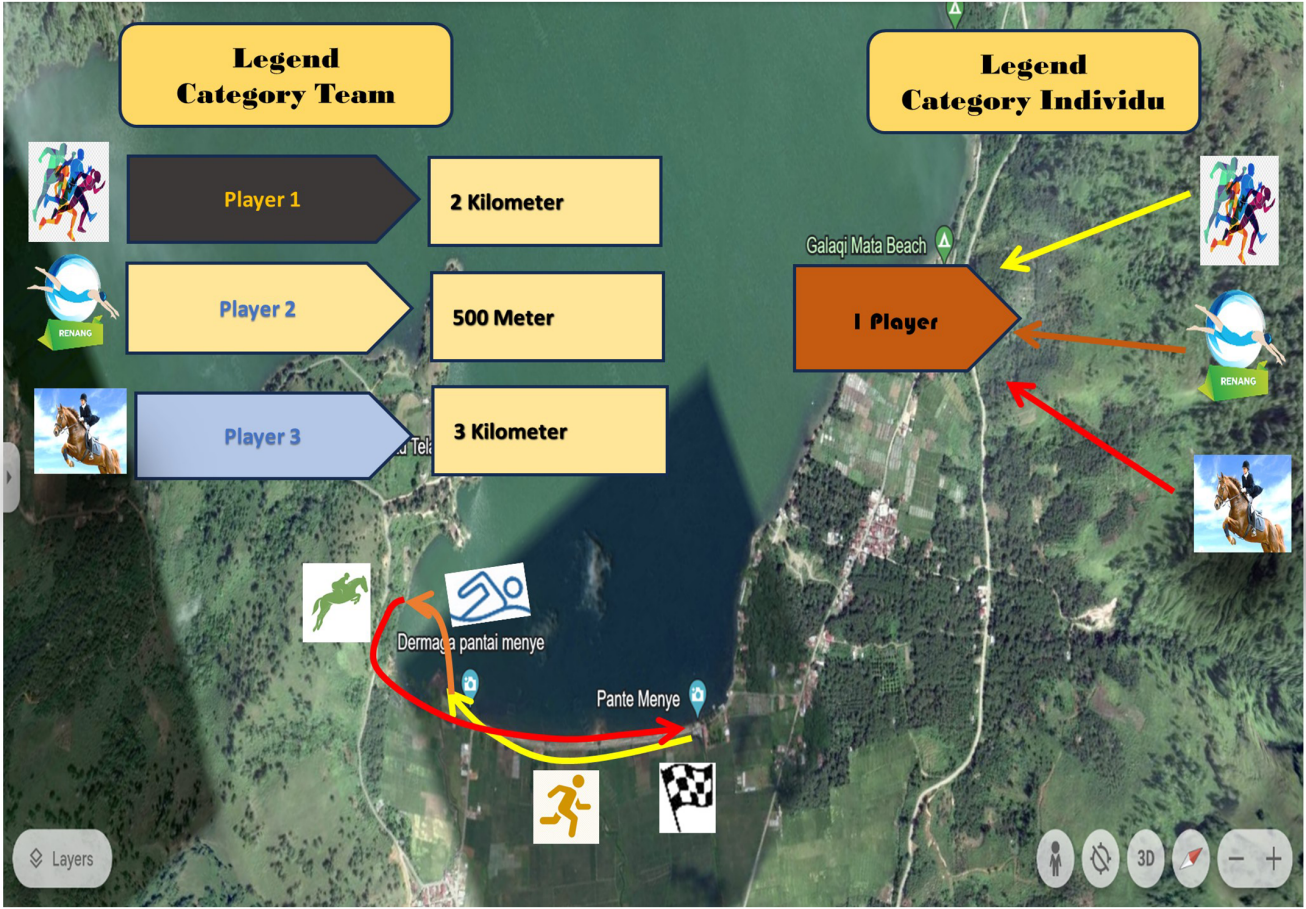
Final product “Run H2O Ride” in Central Aceh District.

Expert 1 is an expert in measurement and evaluation tests from academics. These lecturers gave an average score on product validation of 93.75 by providing input regarding horse racing facilities and infrastructure. Expert 2 is a tourism marketing management expert from the Central Aceh Regency Culture and Tourism Office who also directly conducted field trials with an average value of 91.66 and provided input for the route so that the audience could watch the entire race in one area and not far from each other. Expert 3 is a recreational sports expert from the Youth and Sports Office, Central Aceh Regency, who participated in the field trial with an average score of 89.58. Expert 4 is an expert in the sport of Triathlon from Aceh Province and provided input on the route and mileage of the predetermined “Run H2O Ride” development model, giving an average score of 93.75. The product was revised after experts validated the new model of sports tourism, “Run H2O Ride”. The end product of a new sports tourism model, “Run H2O Ride”, includes team and individual race categories, selection of paths taken, distance from running, swimming, and horse-riding categories, and strategic locations in organizing events. This can be seen in Figure 3.

Then, 20 respondents carried out external validation of the research data. Based on the validity of the instrument test using the moment product correlation test, it was found that the r count ranged from 0.272–0.503, the calculated R-value when compared to the 5% significant level r table, which is 0.254, then the calculated R-value was greater than the r table so the instrument was said to be valid and could be used to assess research products. The reliability of the study was measured directly using the Cronbach alpha reliability test (Table 4). Cronbach's alpha value for expected social impact factor was recorded to be above the recommended 0.70 threshold (148, 149).

**Table 4:** Reliability statistics.

Cronbach's alpha	*N* of Items
0.723	10

Source: own elaboration.

### 4.1 The effectiveness of the new model of sports tourism “Run H2O Ride”

**Figure 4:**
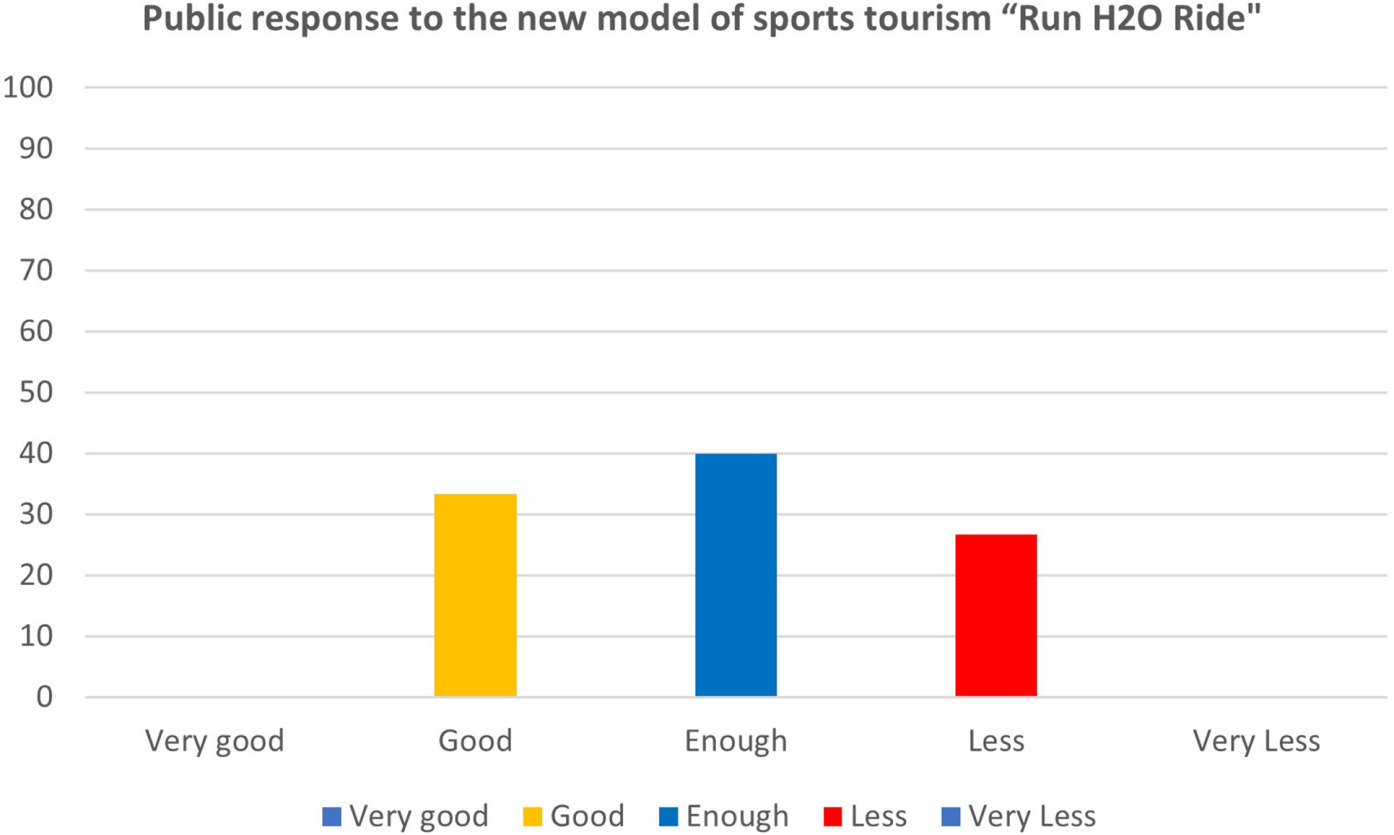
Scores of limited product test “Run H2O Ride”.

Then, three main tests were conducted: limited product test, main product test, and operational product test, to help determine the suitability and effectiveness of this new sports tourism model, which can be seen in Table 5. To assess the suitability and effectiveness of this new sports tourism model, we began the research with a sample size of 15 respondents. Subsequently, three main tests were carried out: product testing was limited to the “Run H2O Ride” product. The public response to the model was rated 33.3% good, 40% enough, and 26.7% less, as shown in Figure 4. The assessment results of the quantitative analysis study show that the sports tourism model product “Run H2O Ride” must be furnished with guidelines for implementation in the form of race regulations. Improving research products should also involve participation from local communities and tourists.

**Table 5:** Public response to the new model of sports tourism “Run H2O Ride”.

No.	Field test	Response	Criterion (%)				
			Very good	Good	Enough	Less	Very less
1.	Limited product test	15 people	0	33.3	40	26.7	0
2.	Main product test	30 people	73.3	20	6.7	0	0
3.	Operational product test	60 people	86.6	11.7	1.7	0	0

Source: own elaboration.

**Figure 5:**
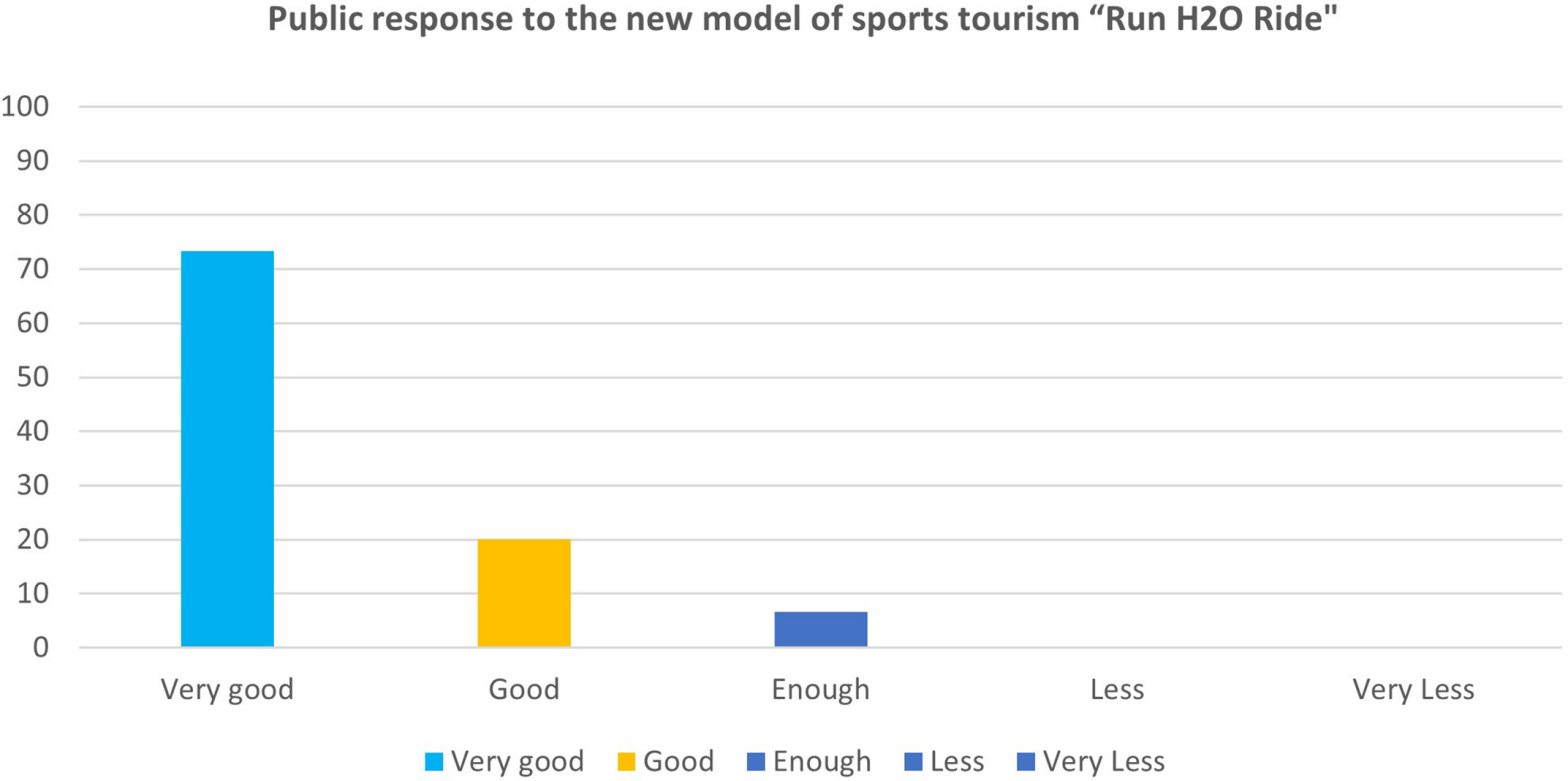
Scores of main product test “Run H2O Ride”.

The main product trial phase was conducted with 30 respondents. The public response to the model was rated 73.3% very good, 20% good, and 6.7% enough, as shown in Figure 5. The assessment results of the quantitative analysis study show that the sports tourism model product “Run H2O Ride” needs to be equipped with a high level of safety for race participants, such as ambulance vehicles and first aid stations. In addition, it is necessary to create a stand for visitors to watch the game and a rest area for participants.

**Figure 6:**
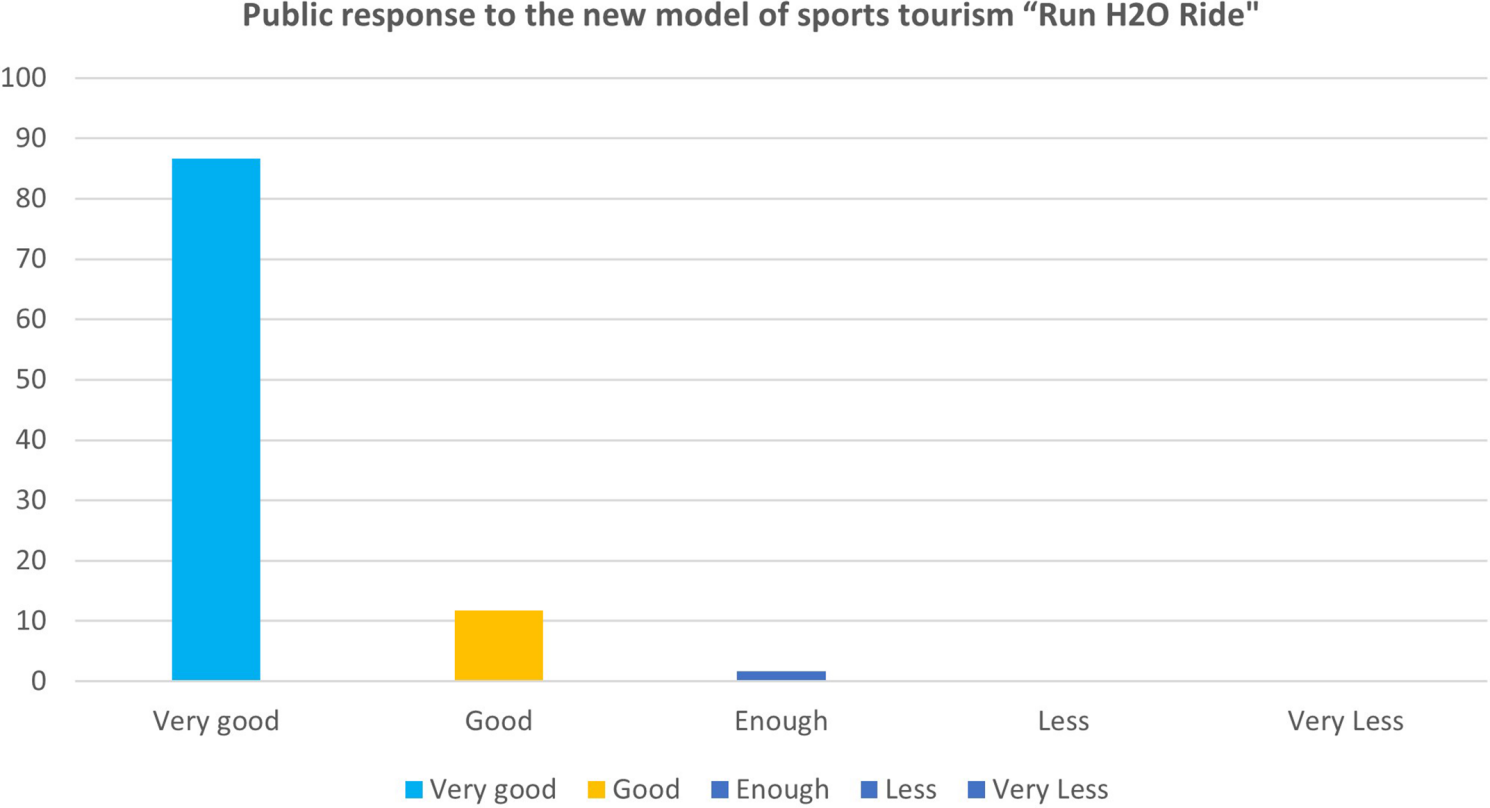
Scores of operational product test “Run H2O Ride”.

The operational product test was the last stage and it had 60 respondents. The public response to the model was rated 86.6% very good, 11.7% good, and 1.7% enough, as shown in Figure 6. The assessment results of the quantitative analysis study show that the new sports tourism model product “Run H2O Ride” has been declared very good and is ready to be used in the implementation of the “Tour Delut Tawar” event in the next implementation. The analysis results indicate that the sports tourism model product ‘Run H2O Ride’ in Central Aceh Regency is compatible with the intended product and is effective for its intended use. In addition, it fulfills the research objectives.

## 5 Discussion

The findings show that the new sports tourism model combined with the local community sports heritage in terms of suitability and effectiveness is very good for influencing the decision of prospective tourists to visit an area that has many natural tourism destinations. Every region of Indonesia has its own cultural traditions and natural resources, giving the country its unique beauty and attracting tourists from around the world (150). This location's primary tourist attractions are its diverse natural and cultural attractions (151, 152). The presence of sports heritage in the sports tourism industry serves as a strategic step to highlight a region's unique characteristics. The success of sports tourism activities is associated with sports heritage and getting support from local communities; hence, local governments must consider community participation in these activities (153). Giango et al. (11) state that sports tourism is widely regarded as an essential form of tourism that draws tourists, improves the local economy, and promotes culture. Thus, the community is very important in tourism development and planning (154, 155). For the Gayo tribe, horse riding is a characteristic of the local people because of the history and geography of this area, which is in the mountains, so horses became a means of transportation for the community.

In addition, the region has a combination of mountains, lakes, rivers, forests, and beaches, creating a diverse and fascinating natural beauty to explore. The attractiveness of natural resources in a tourist area location is the most important factor creating a sense of interest for visitors to visit tourist sites to carry out sports tourism activities (151, 156). Meanwhile, Su et al. (157) emphasize that triathlon sports are very good to be held in a place with many natural attractions. Based on this, one way to increase tourist visits is by combining sports heritage with triathlon sports. The result of this combination created a new sports tourism product: running, swimming, and horse riding. Thus, the targets and results of organizing the upcoming “Tour Delut Tawar” event are hoped to be achieved.

No less important is to provide a sense of security and comfort for visitors who come, by creating a good image for the area. Risk perception greatly influences a traveler's decision to travel somewhere (158, 159). Risk perception of a particular country, especially in the context of destination image studies (160), is significant. It is generally believed that factors influencing the return visit are destination image, quality, perceived destination value, and high satisfaction (161–164) thus leading to positive word of mouth (WOM) and customer return visits, which in turn can affect the economic growth of local communities in the tourism industry (165). In addition, the target of the long-term development plan of Aceh Province 2023–2027 can be realized by creating new sports tourism products.

## 6 Conclusions

The new sports tourism model named “Run H2O Ride” is an excellent strategy for sustainable tourism as an initial illustration for new regions in Indonesia in terms of organizing sports tourism events. This new product's success depends on the local government's role in implementing local regulations by embracing local communities in tourism development. In the future, organizing events with models like this will likely be successful, where the synergy between local governments, entrepreneurs, and the community will be based on them having the same view on building tourism so that tourists will be well received and feel welcomed. This positive impact allows tourists to blend in with the community, share traditions, cultures, and lifestyles, and integrate natural charm into their activities.

The results of this study can still be refined with further research based on the limitations of this study; future research can examine more deeply the rules and regulations that are standard for this new sports tourism model, which needs to be done. The research will contribute to providing information on whether this product can be used for the competitive purpose of achieving success for elite athletes. In addition, research related to this model can also be modified with sports heritage in other areas. The implementation of future research can develop tourism promotion products based on modern technology, such as virtual tour reality, which is recommended as an empirical tool that positively impacts tourists’ decisions to visit and participate in the events held. Therefore, the development of new sports tourism products must be an issue that is studied and paid attention to constantly by practitioners and researchers so that sustainable tourism in Indonesia can be implemented evenly in each region.

### 6.1 Theoretical and practical implications

The results of the study have theoretical and practical implications. This research fills the gap in the literature regarding the implementation of sports tourism events for new regions that want to promote tourism through sports tourism activities. Our findings show that a new sports tourism event we named “Run H2O Ride” can play a significant role in attracting visitors, admittedly providing a new foundation for sustainable tourism theory. Furthermore, practical implications include alternative solutions to address problems that can be implemented to increase tourist visits, with the local governments encouraged to prioritize the characteristics or uniqueness of the sports heritage of the local community. One approach is adopting the sports tourism model “Run H2O Ride,” which is proven to have a positive impact on the current interest in tourist behavior in visiting new places. In addition, we recommend that the local government of Central Aceh Regency, in this case, the Youth and Sports Office and the Tourism Office, make policies for the implementation of future sports tourism events and create a “Run H2O Ride” event. Concomitantly, it can help realize the Aceh Provincial Government's Long-Term Development Plan 2023–2027.

## Data Availability

The original contributions presented in the study are included in the article/**Supplementary Material**, further inquiries can be directed to the corresponding author.
