# Supplementary material for: New model of sports tourism with sustainable tourism development to increase tourist arrivals in Central Aceh Regency, Indonesia
Source: Front Sports Act Living. 2024 Jul 11;6:1421363. doi: 10.3389/fspor.2024.1421363 (PMC11269159; doi:10.3389/fspor.2024.1421363)
Supplement: Supplementary file 1 [file Table1.docx]

Supplementary Material

# Supplementary Data

Supplementary Material should be uploaded separately on submission. Please include any supplementary data, figures and/or tables.

Supplementary material is not typeset so please ensure that all information is clearly presented, the appropriate caption is included in the file and not in the manuscript, and that the style conforms to the rest of the article.

# Supplementary Figures and Tables

For more information on Supplementary Material and for details on the different file types accepted, please see [here](https://www.frontiersin.org/guidelines/author-guidelines#supplementary-material).

## Supplementary Figures

**
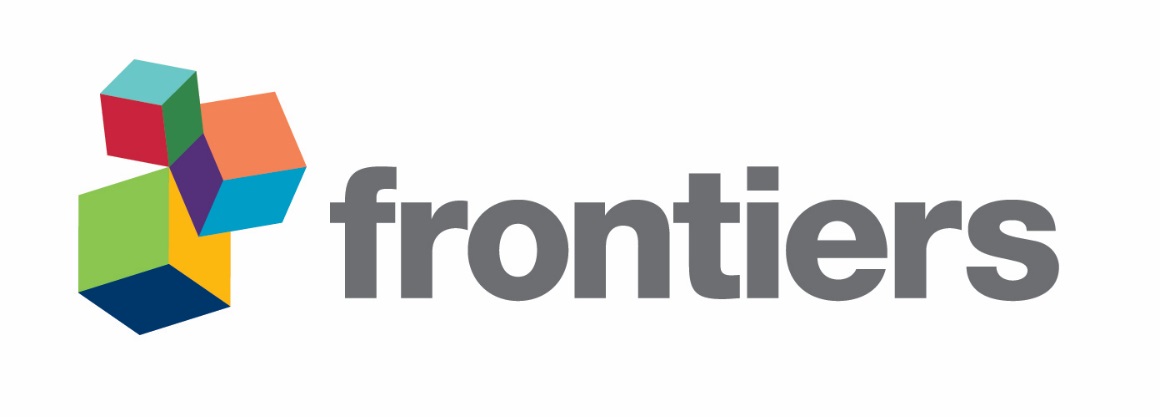
**

**Supplementary Figure 1.** The figure legends are required to have the same font as the main text, 12 point normal Times New Roman, single spaced. Please use a single paragraph for each legend and prepare the figures keeping in mind the PDF layout.

**New Model Sport Tourism Product Race Rules "Run H2O Ride".**


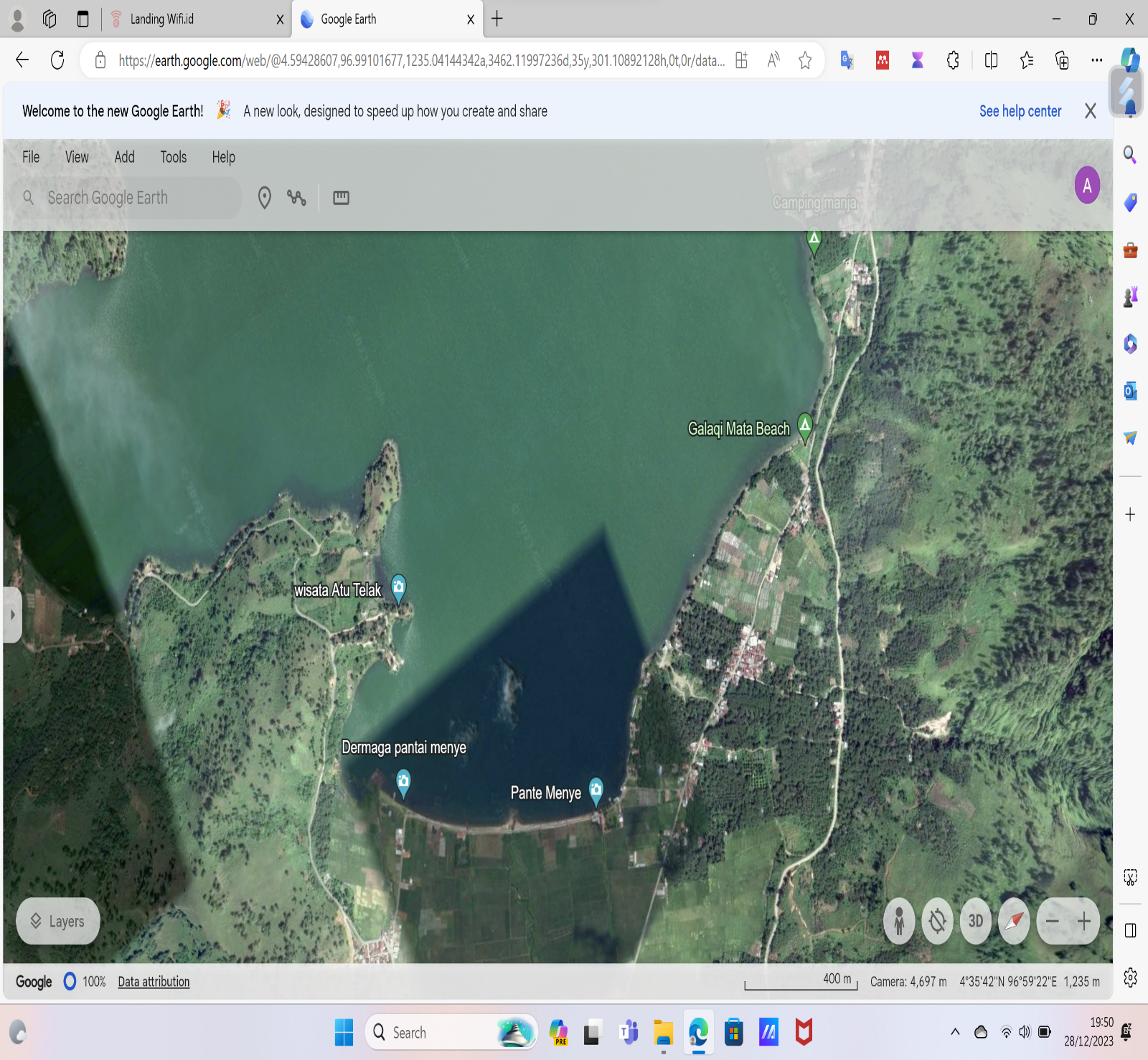

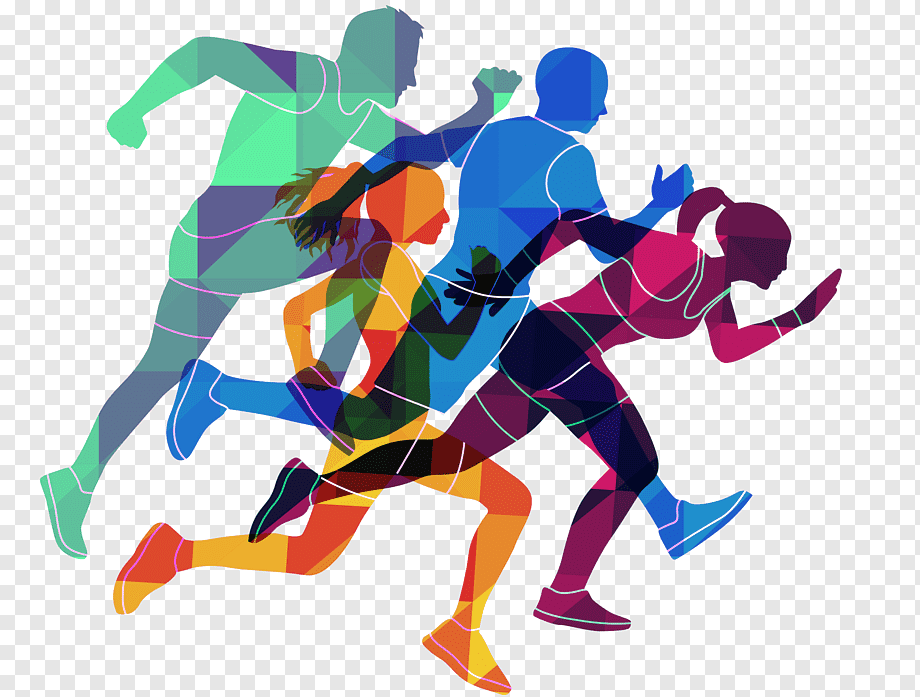

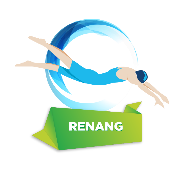

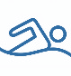

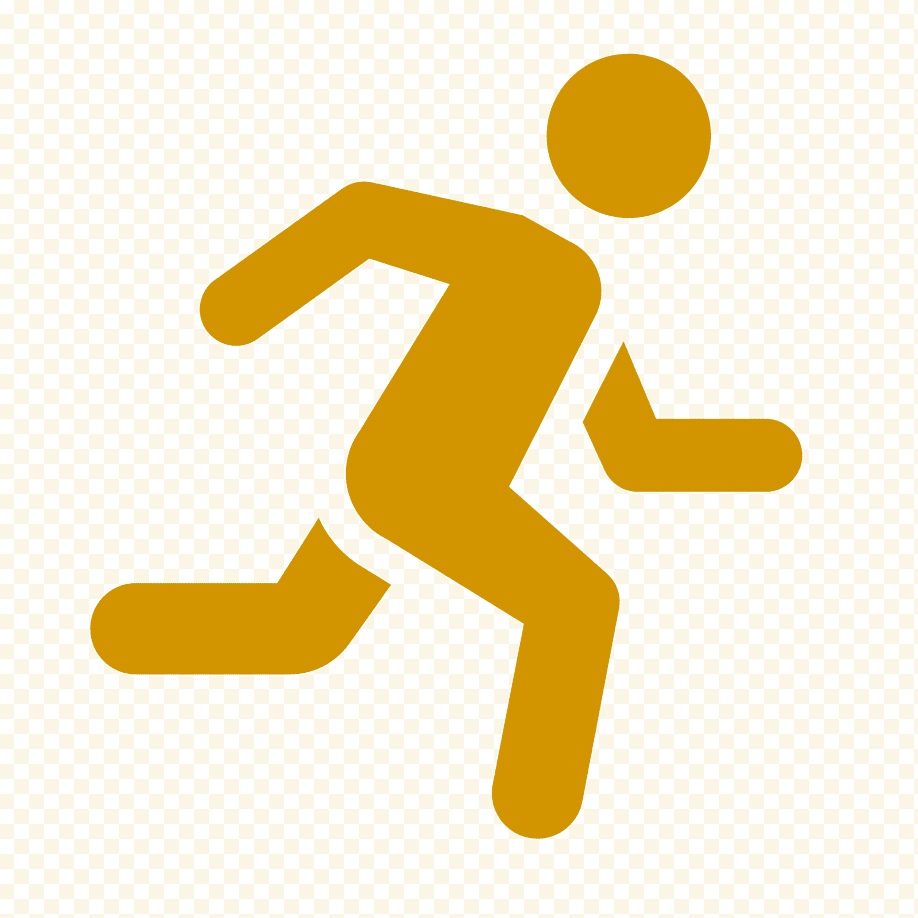

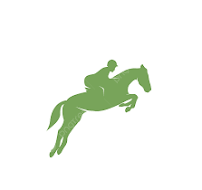


**Player 1**

**Player 2**

**Player 3**


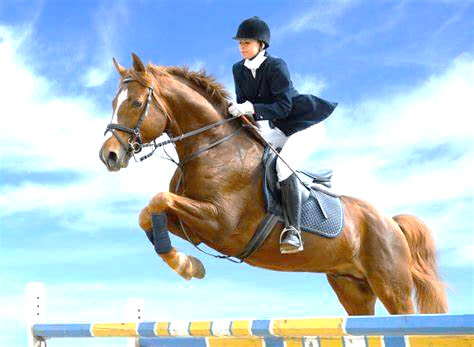


**Legend**

**Category Team**

**Legend**

**Category Individu**


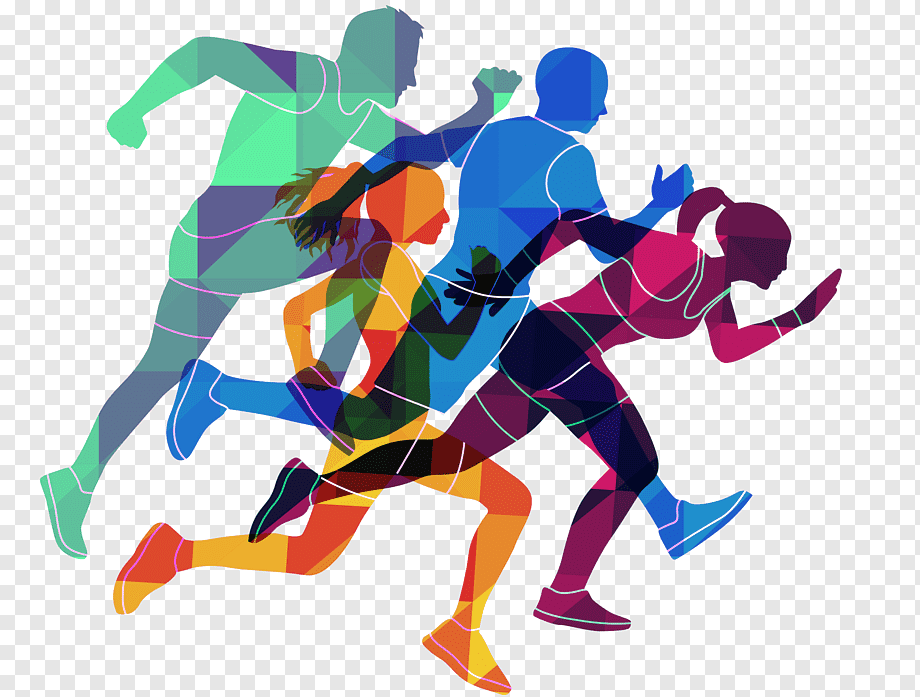


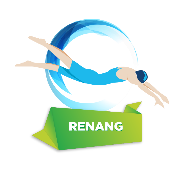


1 Player


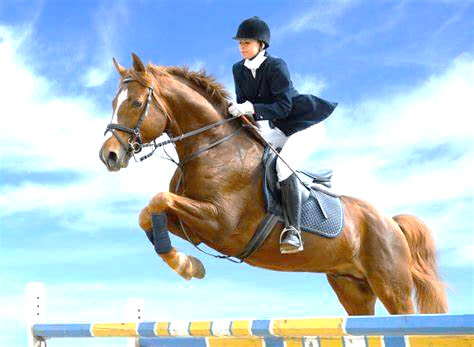


**Produk 1: Rute “Run H20 Ride” Team dan Individu**

Participants of the sports tourism competition "Run H20 Ride" consist of team participants and individual participants. For team participants consisting of 3 people, each participant made the initial starting line of the race starting from "Run" (running) as far as 2 kilometres from the Pante Menye tourist site to the Menye Beach pier. Then, after arriving at the Lake Luttawar area, participants carried out the second sports activity, namely "H2O" (Water), where participants swam with a straight route stretching (in the same direction) as far as 600 meters. Furthermore, the last participant carried out a sports activity, "Ride" (riding), where participants raced on horseback as far as 4 kilometres back to Pante Menye.

Special rules for team participants consisting of 3 people, each competing according to the order in which the race is carried out or in the sense of a race in relay running. One of his friends must finish by reaching the finish line on each route to be able to rotate with his team; rotation is done by patting the body part of the team. Team participants continue to do sports activities in a full series of "Run H2O Ride". However, each session is given the opportunity to do a rest time in order to prepare everything in the race. This competition uses the starting line simultaneously for both team and individual participants. The starting line starts from Pante Menye, and the finish line ends at Pante Menye. For a clearer review, you can see the Sports Tourism regulation chart "Run H2O Ride" below:

**Table 1: "Run H2O Ride" Team Participant Rules Design**

| Team Participant Rules | Sports Activities | Conditions |
| --- | --- | --- |
| 1. Each team consists of 3 participants, and there are three sessions in this competition. 2. Sequentially, session 1 is session "Run", session 2 is "H2O", and session 3 is "Ride". 3. The rules of the team participant competition resemble a relay running match where each team must complete according to the sequence of sports tourism activities "Run H2O Ride", which has been determined by its route so that it can rotate with its team to be able to compete to the finish line. 4. During the rotation, the committee recorded the time of team participants in carrying out sports tourism activities, "Run H2O Ride", in each session. 5. Team participants are given a break to rest when competing so that they can prepare to run the starting line simultaneously with other participants. 6. The match committee determines winners by calculating the total number of fastest time records from each "Run H2O Ride" route collected by all team participants. | **Run** | Team participants started and ran as far as 2 kilometres from the Pante Menye tourist site to the Menye Beach pier. |
|  | **H2O** | Team participants swim with a straight route stretching (in the same direction) as far as 600 meters. |
|  | **Ride** | Team participants race on horseback for 5 kilometres and return to the finish line to Pante Menye. |

In the development of the "Run H2O Ride" Sports Tourism Event, special rules were also made for individual participants who are interested in carrying out the entire series of "Run H2O Ride" activities alone from start to finish based on the race category. The table below reviews the regulations.

**Table 2: Design of "Run H2O Ride" Individual Participant Rules**

| Team Participant Rules | Sports Activities | Conditions |
| --- | --- | --- |
| 1. Individual participants are divided into five competition categories. 2. Individual Participants "Run" 3. Individual Participant "H2O" 4. Individual Participant "Ride" 5. Individual Participants by doing two sports tourism activities.  - example: “Run – H2O”, and “H2O- Ride”  1. Individual Participants "Run H2O Ride" 2. There are three consecutive competition sessions: session 1 is "Run," session 2 is "H2O," and session 3 is "Ride." 3. The competition rules for individual participants, "Run H2O Ride", complete according to the order in which the route has been determined based on the race category from the starting line to the finish line. 4. In each session, the committee records the time that individual participants carry out "Run H2O Ride" activities. 5. Individual participants are given a break to prepare for the starting line simultaneously with other participants. 6. The calculation of winners is determined by the calculation of time records or the total number of fastest time records recorded by the race committee "Run H2O Ride" based on the category participated by individual participants. | **Run** | Participants started and ran as far as 2 kilometres from the Pante Menye tourist site to the Menye Beach pier. |
|  | **H2O** | Participants swim a straight route stretching (in the same direction) as far as 600 meters. |
|  | **Ride** | Participants race on horseback for 4 kilometres and return to the finish line to Pante Menye. |

Description of race numbers for individual participants "Run H2O Ride" by category consists of 5 race categories.

- - 1. Individual participants "Run" are participants who only participate in running competitions without participating in other series of competitions or in the sense that participants only follow running activities as far as 3 kilometres from the Pante Menye line (starting line) to Pante Menye Pier (Finish).
    2. Individual Participant "H2O" is a participant who only participates in a swimming competition without participating in a series of other competitions or in swimming activities as far as 600 meters from a straight (unidirectional) route.
    3. Individual Participant "Ride" is a participant who only participates in an equestrian race without participating in another series of races or in the sense that participants only do equestrian activities as far as 4 kilometres back to the finish line to Pante Menye.
    4. Individual participants doing two sports tourism activities such as: "Run – H2O" and "H2O- Ride", are participants who take part in 2 series of sports tourism activities alone.
    5. Individual Participants "Run H2O Ride" are participants who take part in the entire series of running, swimming and riding competitions alone or in the sense that participants follow running activities as far as 2 Kilometers from Pante Menye to Pante Menye Pier, then continue to swim as far as 600 Meters from the route made straight (in the same direction) and finally do horse riding activities as far as 4 Kilometers back to the finish line to Pante Menye.


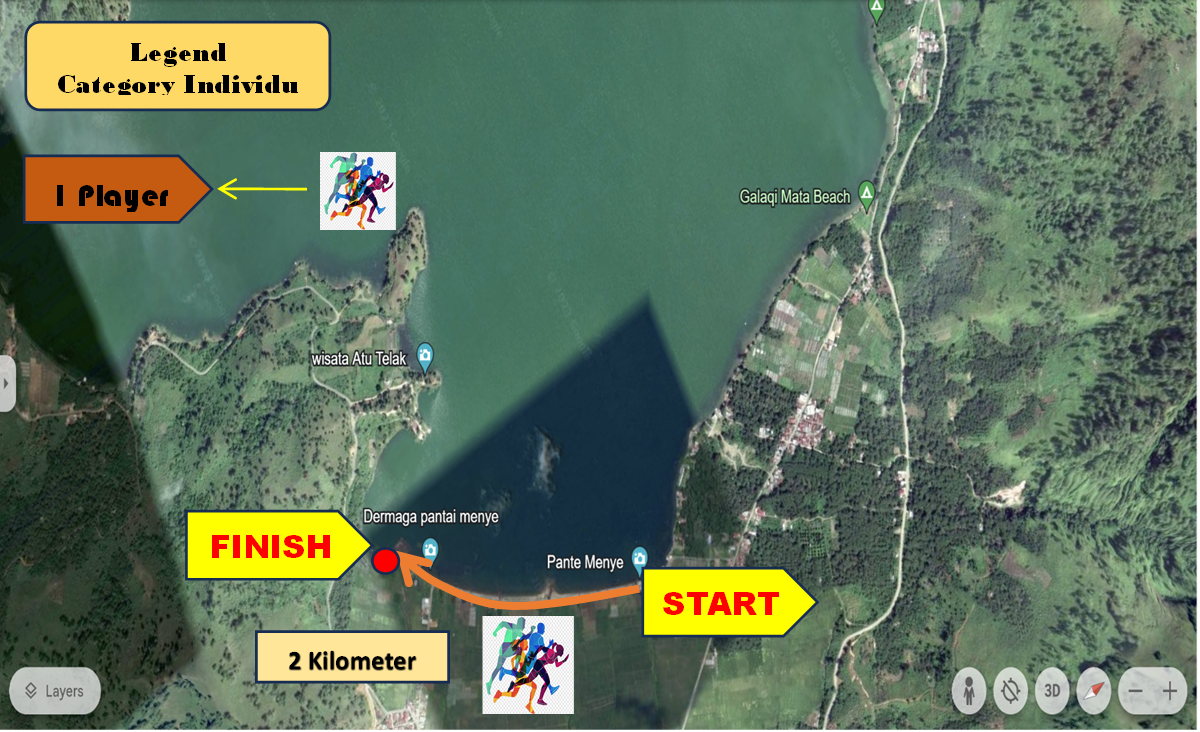
 Thus, the initial design of the route or path from the Sports Tourism model "Run H2O Ride" can be arranged sequentially per item or based on the race category, which can be seen in the explanation of the figure below

**Product 2: Run Route**

In the picture above, it can be seen that " Run H2O Ride" participants can choose sports tourism activities according to the race item of interest or according to one of the race categories, such as the race category only "Run" or running. "Run" participants (only running) start running at the starting line at the pante location at the same time as other race participants who choose items or race categories that also have elements of "Run" or running. Participants ran as far as 2 kilometres to the finish line, namely at the location of the Pante Menye Pier. When at the finish line, the race committee records the travel time obtained by each participant to later be recapitulated by the "Run" race committee.

**
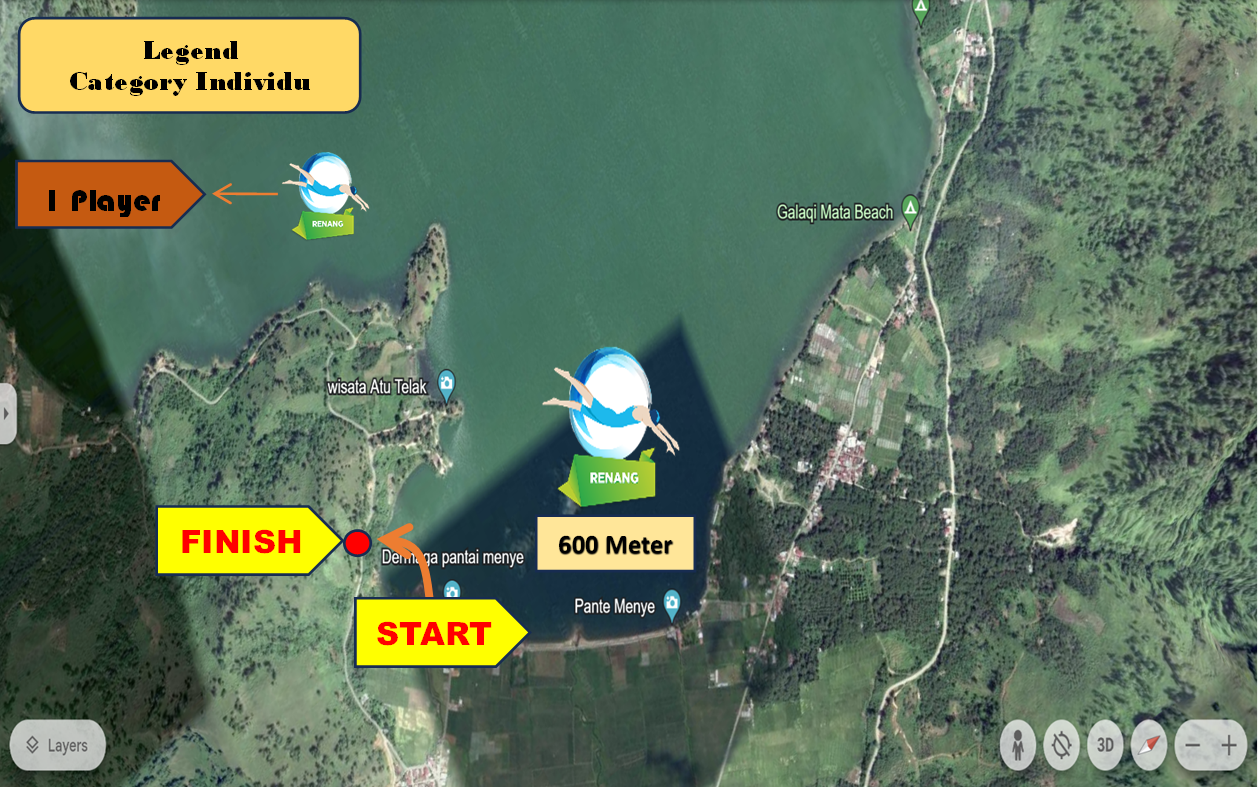
**

**Product 3: H2O Route (Swimming)**

In the picture above, it can be seen that "Run H2O Ride" participants can choose sports tourism activities according to the race item of interest or according to one of the race categories, such as the race category only "H20" or swimming. Participants "H20" (swimming only) start at the starting line at the same time as other race participants who choose items or race categories that also have elements of "H20" or swim from the pante menye pier and then swim as far as 600 meters to the finish line. "H20" participants swim in the Lake Luttawar area with a slightly curved track but remain on the swim track in one direction. For the style of swimming, one is allowed to use any swimming style. Participants are required to wear swimming equipment in accordance with the committee's requirements. When at the finish line, the race committee records the travel time obtained by each participant to later be recapitulated by the "H2O" race committee.


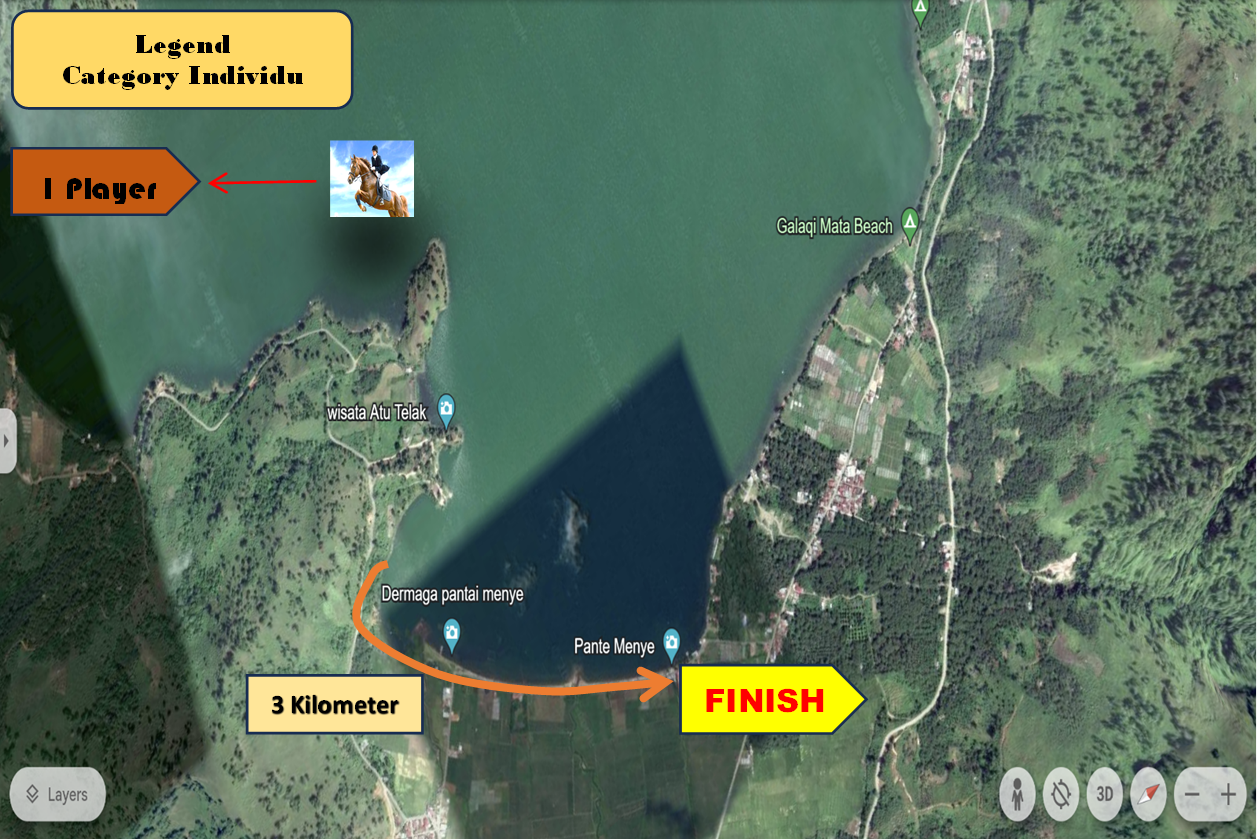


**Product 4: Ride Route (horse riding)**

In the picture above, it can be seen that "Run H2O Ride" participants can choose sports tourism activities according to the race item of interest or according to one of the race categories, such as the race category only "Ride" or horse riding. "Ride" participants (riding only) start at the starting line at the same time as other race participants who choose items or race categories that also contain elements of "Ride". Where participants then race horses as far as 4 kilometres to the finish line back to the pante money. Ride" participants ride on the edge of Lake Luttawar with a slightly curved track but remain on the horse track in one direction. Participants are required to wear equestrian equipment in accordance with the committee's requirements. When at the finish line, the race committee records the travel time obtained by each participant, which will later be recapitulated by the "Ride" race committee.


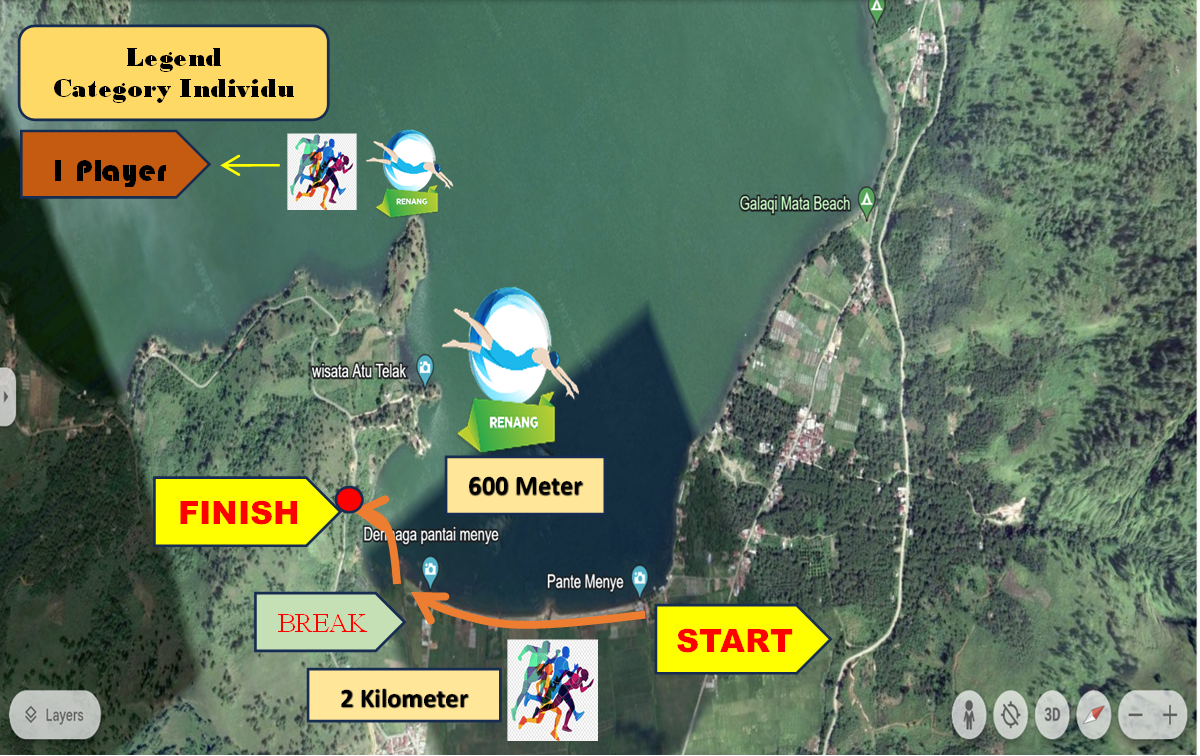


**Product 5: "Run and H2O" Route (Running and Swimming)**

In the product design picture above, it can be seen that the number of participants can also choose race items or categories by following two routes: "Run and H2O (Running and Swimming). In this race number, participants start at the starting line from "Run" along with other participants who follow the elements of the "Run" competition; participants run from the pante menye to the finish line at the pante menye pier. Then, the competition committee "Run H2O Ride" recorded the time of each participant "Run and H2O". from the accumulated amount of time collected in the entire "Run and H2O" race.

Furthermore, participants are allowed to rest while preparing for the next competition activity, namely "H2O" or swimming. Participants started at the starting line at the same time as other participants who also participated in the "H2O" race element by swimming as far as 600 meters in the Lake Luttawar area with a slightly curved track but remained on the swimming track in one direction. For the style of swimming, one is allowed to use any swimming style. Participants are required to wear swimming equipment in accordance with the committee's requirements. When at the finish line, the race committee records the travel time obtained by each participant to later be recapitulated by the race committee "Run H2O Ride". The winner in this category is seen


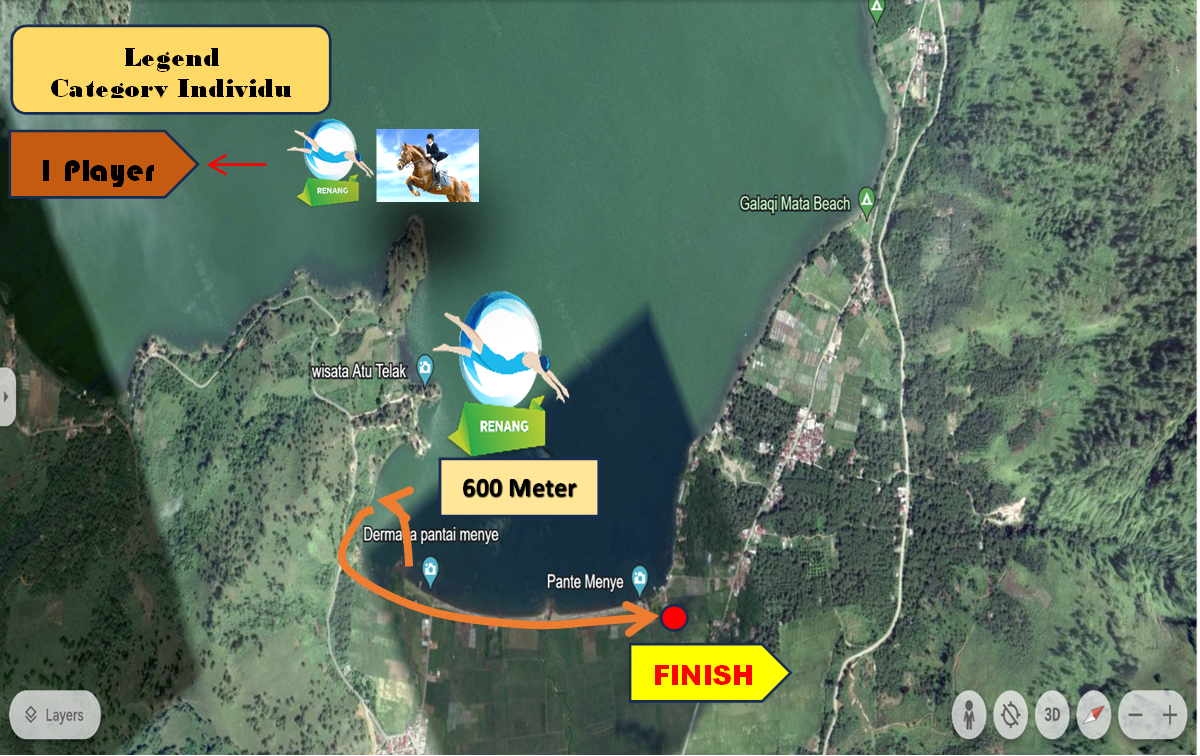
**Product 6: "H2O and Ride" Route (Swimming and Riding)**

In the product design picture above, it can be seen that the number of participants can also choose items or race categories by following two routes, "H2O and Ride" (Swimming and riding). In this race number, participants start the starting line from "H2O" along with other participants who follow the elements of the "H2O" race. Participants swam from the pier panty money. Then, swim 600 meters to the finish line. "H20" participants swim in the Lake Luttawar area with a slightly curved track but remain on the swim track in one direction. For the style of swimming, one is allowed to use any swimming style. Participants are required to wear swimming equipment in accordance with the committee's requirements. When at the finish line, the race committee records the travel time obtained by each participant to later be recapitulated by the "H2O Ride" race committee.

Furthermore, participants are allowed to rest while preparing for the next race activity, namely "Ride". Then, participants start at the starting line together with other race participants who choose items or race categories that also contain elements of "Ride". Where participants then race horses as far as 4 kilometres to the finish line back to the pante money. Ride" participants ride on the edge of Lake Luttawar with a slightly curved track but remain on the horse track in one direction. Participants are required to wear equestrian equipment in accordance with the committee's requirements. When at the finish line, the race committee records the travel time obtained by each participant to later be recapitulated by the "H2O Ride" race committee. The winner in this category is seen from the accumulated amount of time collected in the entire "H2O Ride" race.
